# Clinical characterization of a novel *RAB39B* nonstop mutation in a family with ASD and severe ID causing RAB39B downregulation and study of a *Rab39b* knock down mouse model

**DOI:** 10.1093/hmg/ddab320

**Published:** 2021-11-11

**Authors:** Maria Lidia Mignogna, Romina Ficarella, Susanna Gelmini, Lucia Marzulli, Emanuela Ponzi, Alessandra Gabellone, Antonia Peschechera, Massino Alessio, Lucia Margari, Mattia Gentile, Patrizia D’Adamo

**Affiliations:** Molecular Genetics of Intellectual Disability, Division of Neuroscience, IRCCS San Raffaele Scientific Institute, 20132, Milan, Italy; Medical Genetics Unit, Department of Reproductive Medicine, ASL Bari, 70132, Bari, Italy; Molecular Genetics of Intellectual Disability, Division of Neuroscience, IRCCS San Raffaele Scientific Institute, 20132, Milan, Italy; Child Neuropsychiatry Unit, Department of Biomedical Sciences and Human Oncology, University of Bari “Aldo Moro”, 70126, Bari, Italy; Medical Genetics Unit, Department of Reproductive Medicine, ASL Bari, 70132, Bari, Italy; Child Neuropsychiatry Unit, Department of Biomedical Sciences and Human Oncology, University of Bari “Aldo Moro”, 70126, Bari, Italy; Child Neuropsychiatry Unit, Department of Biomedical Sciences and Human Oncology, University of Bari “Aldo Moro”, 70126, Bari, Italy; Proteome Biochemistry, Center for Omics Sciences, IRCCS San Raffaele Scientific Institute, 20132, Milan, Italy; Child Neuropsychiatry Unit, Department of Biomedical Sciences and Human Oncology, University of Bari “Aldo Moro”, 70126, Bari, Italy; Medical Genetics Unit, Department of Reproductive Medicine, ASL Bari, 70132, Bari, Italy; Molecular Genetics of Intellectual Disability, Division of Neuroscience, IRCCS San Raffaele Scientific Institute, 20132, Milan, Italy

## Abstract

Autism spectrum disorder (ASD) and intellectual disability (ID) often exist together in patients. The *RAB39B* gene has been reported to be mutated in ID patients with additional clinical features ranging from ASD, macrocephaly, seizures and/or early-onset parkinsonism. Here, we describe a novel *RAB39B* nonstop mutation [Xq28; c.640 T > C; p.(^*^214Glnext^*^21)] in a family with ASD, severe ID and poor motor coordination, and we assessed the pathogenicity of the mutation. A heterologous cell system and a *Rab39b* knockdown (KD) murine model, which mimic the nonstop mutation, were used to validate the deleterious effect of the *RAB39B* mutation. The mutation led to RAB39B protein instability, resulting in its increased degradation and consequent downregulation. Using a *Rab39b* KD mouse model, we demonstrated that the downregulation of RAB39B led to increased GluA2 lacking Ca^2+^-permeable AMPAR composition at the hippocampal neuronal surface and increased dendritic spine density that remained in an immature filopodia-like state. These phenotypes affected behavioural performance in a disease-specific manner. *Rab39b* KD mice revealed impaired social behaviour but intact social recognition. They also showed normal anxiety-like, exploratory and motivational behaviours but impaired working and associative memories. In conclusion, we found a novel *RAB39B* nonstop variant that segregated in a family with a clinical phenotype including ID, ASD and poor motor coordination. The pathogenicity of mutations causing the downregulation of RAB39B proteins, impacting AMPAR trafficking and dendritic spine morphogenesis, reinforced the idea that AMPAR modulation and dendritic spine assets could be considered hallmarks of neurodevelopmental disorders.

## Introduction

Autism spectrum disorder (ASD) is a complex neurodevelopmental disorder characterized by deficits in social communication and interaction and restricted, repetitive patterns of behaviour, interests and activities, as listed in the Diagnostic and Statistical Manual of Mental Disorders, Fifth edition (DSM-V). Individuals with ASD often present with other co-occurring conditions, such as other neurodevelopmental disorders (intellectual disability, attention deficit hyperactivity disorder, developmental coordination disorder), neurological disorders (sleep disturbances, epilepsy, or macrocephaly) or medical conditions (gastrointestinal problems or congenital anomalies) (1–3). The level of intellectual functioning in individuals with ASD is extremely variable, extending from profound impairment to high functioning (4,5). It has been estimated that the prevalence of ASD is 1 in 54 children (6).

The etiopathogenesis of the disorder is described as multifactorial, in which genetic and environmental factors are involved. Studies on families (7) and populations (8,9) showed that the major risk of developing ASD is genetic variations, from rare inherited and *de novo* genetic mutations, including single nucleotide variants, small and large insertions or deletions, and other complex structural variations (10–12). ASD is more frequent in boys than girls, with an approximate ratio of 4:1, strongly suggesting an aetiological role for the sex chromosomes. Several X-linked genes associated with intellectual disability (ID) are also causative for ASD (13). Among them, *RAB39B* is associated with ID in comorbidity with ASD, epilepsy, macrocephaly (OMIM: 300774) and/or early-onset parkinsonism (OMIM: 311510).

RAB39B is a neuronal small GTPase that switches from an inactive GDP-bound to an active GTP-bound state and drives the intracellular vesicular trafficking of GluA2/GluA3 α-amino-3-hydroxy-5-methyl-4-isoxazolepropionic acid receptors (AMPARs) from the endoplasmic reticulum (ER) to the Golgi complex, finally orchestrating their postsynaptic surface expression (14). Mutations leading to the full absence of RAB39B protein expression or increased dosage levels have been described (15–24). The full absence of RAB39B in a *Rab39b* knockout mouse model recapitulated some ASD/ID behavioural characteristics (25).

In the present study, we identified a novel mutation consisting of a nonstop mutation [Xq28; c.640 T > C; p.(^*^214Glnext^*^21)] in two brothers with ASD, severe ID and poor motor coordination. This mutation reduced the protein stability, resulting in increased protein degradation, causing a downregulation of RAB39B protein expression. Taking advantage of a *Rab39b* knockdown (KD) mouse model, we detailed the pathophysiological role of RAB39B from molecular and behavioural points of view. *Rab39b* KD mice showed alterations in AMPAR composition, impaired dendritic spine maturation and deficits in social and cognitive behaviour, phenotypes that are hallmarks of ASD and ID.

## Results

### Clinical features of two brothers with intellectual developmental disorder carrying a novel *RAB39B* nonstop mutation

The family was referred to our Medical Genetics Service by the Territorial Child Neuropsychiatry Service for the genetic evaluation of two siblings diagnosed with ID, ASD and motor problems. Probands II-1 and II-2 were evaluated by us for the first time at 4 years and 8 months and at 2 years and 10 months, respectively. The parents were Caucasian nonconsanguineous; their family history was negative for neurodevelopmental and/or ASDs.

**Figure 1:**
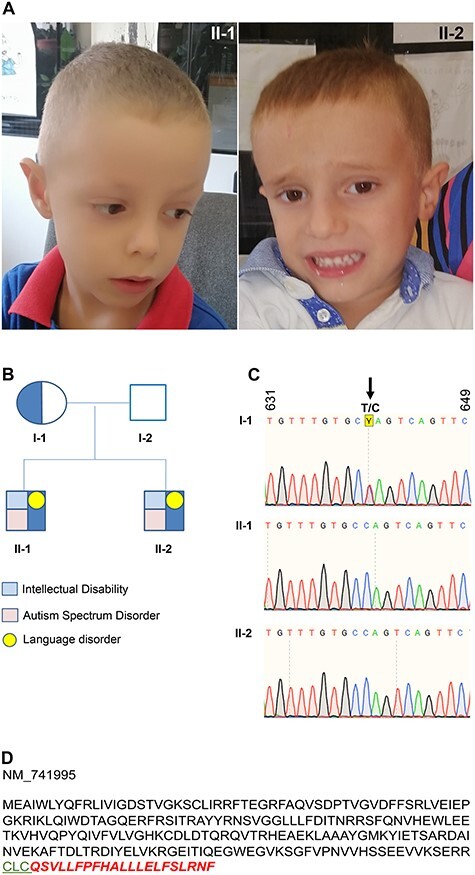
*RAB39B* ter214Q mutation. (A) Probands II-1 and II-2 at 6.6 and 4.7 years of age. **(B)** Pedigree of the family. Circle is female and squares are male. Opened symbols indicate unaffected male and blue indicates the presence of *RAB39B* mutation. Light red and light blue squares and yellow circle specify the type of the disability and/or disorder presented. **(C)** DNA sequence from I-1, II-1 and II-2. Arrows indicate the nonstop mutation changing the TAG stop codon in CAG. Sequence numbers refer to the human *RAB39B* cDNA (NM_171998). **(D)** Human RAB39B protein sequence. In green, the CLC geranyl-geranylation site and in italic bold red the 21 aa tail.

Despite the absence of major dysmorphisms, on physical examination, the similarity in appearance between the two brothers was striking, with a high forehead, large ears, mild generalized hypotonia, and hypotonia of the perioral muscles with drooling and poor motor coordination (Fig. 1A and B).

A detailed neuropsychological evaluation was conducted at the Child Neuropsychiatry Unit of the University of Bari. The last neurologic examination was performed at 6 years and 2 months (proband II-1) and at 4 years (proband II-2). A clear similarity was also present at the neuropsychological level, as summarized in Table 1. A diagnosis of ASD was made in both cases according to the DSM-V criteria and it was supported by the Autism Diagnostic Interview-Revised (ADI-R) and Autistic Diagnostic Observation Schedule (ADOS) tests. Both probands showed a level three severity of social communication and restricted, repetitive patterns of behaviour. A severe deficit in verbal and nonverbal social communication, very limited initiation of social interactions, a minimal response to social overtures from others, great difficulty changing focus or action and inflexibility of behaviour were also present. Receptive and expressive language was significantly impaired. Motor stereotypies such as hand flapping was observed in proband II-2.

**Table 1:** Clinical and neuropsychological evaluation of affected patients. ID: Intellectual disability, ASD: autism spectrum disorder, NA^^*^^: not available, M-P-R: Merill-Palmer-revised, ADI-R: autism diagnostic interview-revised, ADOS: autistic diagnostic observation schedule, VINELAND-II: Vineland Adaptive Behaviour Scales-II

*Clinical evaluation*		
	II-1	II-2
Age	7 years	5 years
Sex	Male	Male
Height	113 cm (50th percentile)	102 cm (50th percentile)
Head circumference	55 cm (90–97th percentile)	54 cm (90–97th percentile)
Neurological examination	Slight generalized hypotonia. Hypotonia of the perioral muscles with drooling poor motor coordination	Light generalized hypotonia. Mild hypotonia of the perioral muscles with drooling motor clumsiness poor motor coordination
Neurodevelopmental disorders	ASD, level 3 Severe Intellectual disability	ASD, level 3 Severe Intellectual disability
Other	Language impairment	Language impairment
*Neuropsychological evaluation*		
	II-1	II-2
Age	6 years and 2 months	4 years
Merill-Palmer-Revised		
*Cognitive battery*		
Developmental index (AE)	45 months	7 months
Cognitive development (AE)	41 months	5 months
Fine motor development (AE)	50 months	7 months
Receptive language (AE)	46 months	NA^*^
Memory (AE)	43 months	NA^*^
Speed (AE)	51 months	NA^*^
Visual motor (AE)	47 months	5/6 months
*Gross motor battery*		
Gross motor	23 months	20 months
*Language battery*		
Expressive language score	28 months	NA^*^
Language score	28 months	NA^*^
*Social-emotion battery*		
Social emotion (parents)	16/17 months	11 months
Self-help adaptive		
Self hel adaptive (parents)	20 months	13/14 months
Vineland-II		
*Communication*		
Deviation IQ	32	30
Adaptive level	low	low
• Receptive	17 months	8 months
• Expressive	11 months	8 months
• Written	47 months	43 months
*Daily living skills*		
Deviation IQ	53	45
Adaptive level	low	low
• Personal	31 months	25 months
• Domestic	42 months	24 months
• Community	42 months	22 months
*Social skills*		
Deviation IQ	45	34
Adaptive level	low	low
• Interpersonal relationship	7 months	11 months
• Play and leisure time	5 months	20 months
• Coping skills	27 months	25 months
*Motor skills*		
Deviation IQ		36
Adaptive level		low
•Gross motor	NA^*^	26 months
• Fine motor	NA^*^	28 months
ADI-R		
	II-1	II-2
Deficits in social interaction (cut-off:10)	18	22
Deficits in social communication (cut-off 8)	13	12
Restricted, repetitive patterns of behaviour, interests, or activities (cut-off 3)	4	7
Developmental deficits before 36 months (cut-off 1)	5	5
ADOS-2 (MOD1)		
	II-1	II-2
Social Interaction	17	19
Stereotype behaviours and restricted interest	6	8
Tot.	23	27
Level of symptoms	High	High

Neurocognitive abilities, including Merill-Palmer-Revised (M-P-R) scales assessment, revealed a developmental index of 45 months (equivalent age 6 years and 2 months, proband II-1) and of 7 months (equivalent age 4 years, proband II-2) with a standardized score of 20 corresponding to severe cognitive impairment. Deficits in adaptive behaviour were assessed using the Vineland Adaptive Behaviour Scales-II (VABS); personal and social autonomies were significantly impaired with a deviation intellectual quotient (IQ) of 35 (proband II-1) and 25 (proband II-2), indicative of a low level of adaptive functioning.

Karyotype, chromosomal microarray, and fragile X testing were performed as first-tier tests for ASDs and were normal. Considering the well-established role of whole exome sequencing (WES) as an efficient diagnostic tool for complex neurodevelopmental phenotypes and/or ASD and the overlapping clinical phenotype of the two brothers of our family, we performed WES analysis.

Autosomal recessive and X-linked inheritance models were queried to investigate a monogenic model in our family. A nonstop mutation in *RAB39B* [Xq28, c.640 T > C; p.(^*^214Glnext^*^21)] was identified. Both probands were found to be hemizygous for the variant, which, as predicted for the X-linked inheritance model, was inherited from their heterozygous mother (Fig. 1B and C). The mutation abolished the canonical stop codon, generating a late stop codon after 63 base pairs (bps), resulting in 21 amino acid (aa) additions to the C-terminus of the RAB39B protein (Fig. 1D).

### The nonstop *RAB39B* mutation, RAB39B ter214Q, affected protein stability, leading to RAB39B downregulation

The C-terminus of RAB GTPases contains the CXC geranylgeranylation site, where geranylation is necessary for tail anchoring to the vesicle membrane, allowing the GTPase to execute its function (26).

The analysis of the known domains and the consensus motifs potentially contained in the 21 aa string by using the major recognized scientific research databases (https://blast.ncbi.nlm.nih.gov/Blast.cgi? PAGE = Proteins; https://www.UniProt.org/;https://prosite.expasy.org/;https://www.ebi.ac.uk/interpro/beta/) did not reveal any significant results, with the exception of 160 hits, identified with low scores by NCBI BLAST, corresponding to transmembrane domains of more than 100 different unrelated proteins. This is likely due to the hydrophobic nature of the additional leucine-rich (L) stretch of aas (Fig. 1D). Interestingly, the secondary structure prediction of the extra 21 aas, performed both with PredictProtein and JPred4 online tools (https://predictprotein.org/home and http://www.compbio.dundee.ac.uk/jpred4/index.html), underlined the alpha-helix organization of the central 17 aa, which in turn suggested a putative transmembrane function. This was supported by the significant scores reached by transmembrane prediction performed with TemPred tools on ExPASy (https://embnet.vital-it.ch/software/TMPRED_form.html).

**Figure 2:**
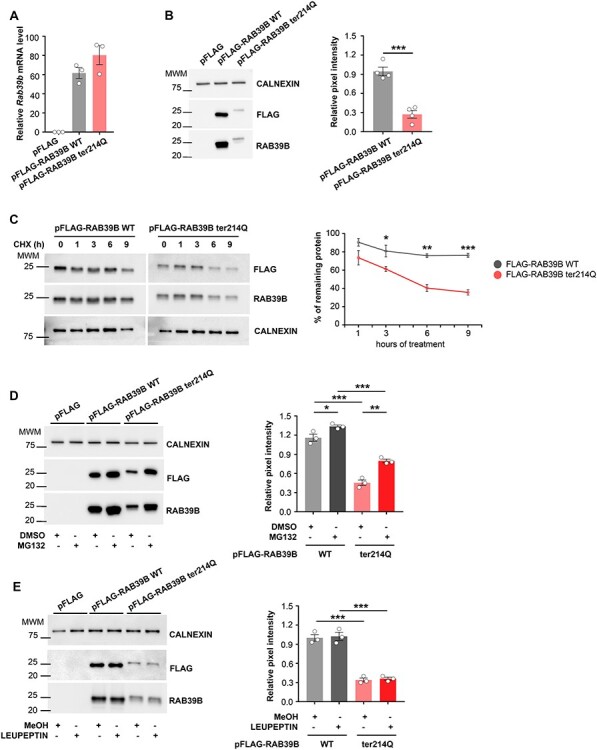
*RAB39B* ter214Q characterization. **(A)** Expression level of *Rab39b* transcript in COS7 cells transfected with pFLAG or pFLAG-RAB39B WT or pFLAG-RAB39B ter214Q (*n* = 3). Data are presented as relative mRNA level of *Rab39b* calculated as 2^-ΔCt(Rab39b–H3)^. **(B)** Representative western blots of COS7 cells expressing pFLAG (1 KDa, no band) or pFLAG-RAB39B WT (24.63 KDa) or pFLAG-RAB39B ter214gln (27.11 KDa) (*n* = 4), CALNEXIN (90 KDa) are used as housekeeping gene. **(C)** Graph indicates RAB39B protein expression normalized on CALNEXIN. **(D)** Representative western blots of COS7 expressing pFLAG or pFLAG-RAB39b WT or pFLAG-RAB39B ter214gln, treated with MG132 or DMSO as control (*n* = 3). CALNEXIN was used as housekeeping gene. **(E)** Graph indicates RAB39B protein expression normalized on CALNEXIN**.** MWM: molecular weight marker of protein ladder. Data are presented as mean ± SEM. Student’s *t*-test was used in **(B, D** and **E)** and two-way ANOVA interaction and Student’s *t*-test was used in (C).^*^P < 0.05, ^*^^*^P < 0.01, ^*^^*^^*^P < 0.001.

Next, to evaluate the impact of the additional 21 aas on RAB39B protein function, we evaluated the *RAB39B* gene and protein expression by transfecting the heterologous COS7 cell system with pFLAG-RAB39B ter214Q or an empty pFLAG plasmid or pFLAG-RAB39B WT, where the last two plasmids were used as controls. By qRT-PCR, a comparable amount of *RAB39B* transcript expression between *RAB39B ter21Q* and *RAB39B WT* was detected (Fig. 2A). In contrast, immunoblotting showed 70% downregulation of FLAG-RAB39B ter214Q protein expression compared to FLAG-RAB39B WT (Student’s *t*-test *P* = 0.0003) and a protein increment of the molecular weight from 24.63 to 27.11 KDa (as predicted by https://www.bioinformatics.org/sms/prot_mw.htm;Fig. 2B), suggesting that the mutated protein was being expressed.

Because of the normal level of the RAB39B RNA messenger, the observed decrease in the RAB39B ter214Q protein amount could depend on protein instability. To examine the effect of the variant on RAB39B stability, the protein turnover rate was analyzed after blocking protein translation with cycloheximide. COS7 cells transfected with pFLAG-RAB39B WT or p-FLAG-RAB39B ter214Q were subjected to cycloheximide chase analysis. Strikingly, RAB39B ter214Q was unstable compared to WT, with a half-life of <3 h, while the WT form was highly stable (two-way ANOVA interaction F[3, 16] = 3.3 *P* = 0.047; FLAG-RAB39B WT vs. ter214Q Student’s *t*-test 3 h *P* = 0.048, 6 h *P* = 0.0014, 9 h *P* = 0.0003; Fig. 2C).

Moreover, the elimination of the RAB39B ter214Q protein could be mediated by the cytoplasmic degradation machinery, proteasome or lysosomes. Thus, MG132 (Fig. 2D), an inhibitor of the proteasome complex, and leupeptin (Fig. 2E), a structurally related lysosome inhibitor, were used (27). COS7 cells were treated with 2 μM MG132 and 20 μM leupeptin after transfection with empty pFLAG or pFLAG-RAB39B WT or pFLAG-RAB39B ter214Q. We showed that the degradation of RAB39B ter214Q was blocked by MG132 (RAB39B ter214 DMSO vs. MG132 Student’s *t*-test *P* = 0.003; Fig. 2D), similar to the WT form (RAB39B WT DMSO vs. MG132 Student’s *t*-test *P* = 0.04; Fig. 2D). In addition, RAB39B ter214Q was more sensitive to MG132 than the WT form (delta RAB39B WT MG132—DMSO 0.17 ± 0.03 vs. delta RAB39B ter214Q MG132—DMSO 0.34 ± 0.02, Student’s *t*-test *P* = 0.01). In contrast, leupeptin did not impact RAB39B ter214Q or WT (Fig. 2E).

These data suggested that the nonstop *RAB39B* mutation adding 21 aas at the C-terminus of the RAB39B protein led to protein instability and that the 70% protein downregulation was caused by protein degradation by the proteasome complex.

### Thirty percent of the RAB39B ter214Q protein executed its AMPAR trafficking role

**Figure 3:**
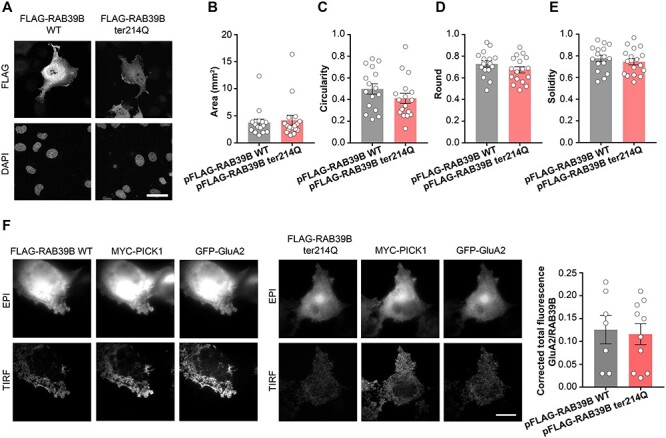
*RAB39B* ter214Q characterization. **(A)** Representative images of COS7 cells transfected with pFLAG-RAB39B WT or p-FLAG-RAB39B ter214Q. Scale bar is 50 μm. **(B—E)** Morphological analysis of transfected COS7 cells. **(F)** Representative TIRF and epifluorescence images of COS7 cells transfected with pFLAG-RAB39B or pFLAG-RAB39B ter214gln together with MYC-PICK1 and GFP-GluA2. Quantification of surface GluA2 related on total RAB39B. Scale bar, 20 μm. Data are presented as mean ± SEM.

We previously reported that RAB39B was involved in transporting the GluA2-AMPA receptor subunit by PICK1 bridging (14). We then evaluated whether the expressed FLAG-RAB39B ter214Q might exert its role in mediating GluA2 AMPAR subunit trafficking toward the plasma membrane in COS7 cells, as previously described (15). First, we demonstrated that the morphological cell integrity of COS7 cells transfected with pFLAG-RAB39B ter214Q was preserved, similar to the pFLAG-RAB39B WT (Fig. 3A–E). Next, COS7 cells were transfected with pFLAG-RAB39B ter214Q or pFLAG-RAB39B WT as a control, together with myc-PICK1 and GFP-GluA2, and we investigated the presence of GFP-GluA2 on the plasma membrane by TIRF (total internal reflection fluorescence) analysis. We showed that GluA2 was trafficked to the plasma membrane when PICK1 was coexpressed with RAB39B WT or RAB39B ter214Q (Fig. 3F). The quantification of TIRF experiment suggests that the remaining 30% of the RAB39B ter214Q protein was sufficient to drive the GluA2 subunit to the plasma membrane (Fig. 3F, right panel).

### The downregulation of RAB39B protein impacts AMPAR trafficking and spine maturation, leading to social and cognitive deficits in a *Rab39b* knock down mouse model

Taking advantage of the development of *Rab39b* knockout mice, where the complete absence of RAB39B affected AMPAR trafficking, increasing synaptic network excitability, which correlated with immature spine development and behavioural and cognitive deficits in adult mice (25), we wondered whether the downregulation of RAB39B could generate a similar phenotype. We used a *Rab39b* knockdown (KD) mouse model generated in our laboratory, with a significant downregulation of RAB39B protein expression.

**Figure 4:**
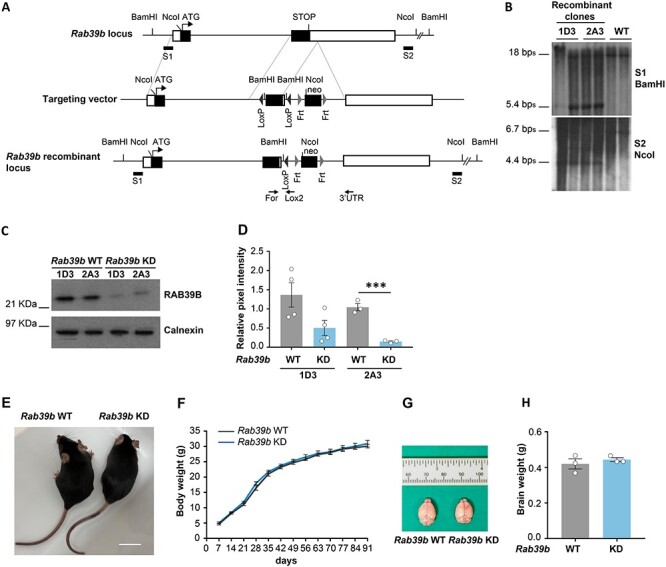
Generation and characterization of *Rab39b* KD animal model. **(A)** Scheme of the structural organization of the *Rab39b* locus where exons are black boxes spaced out from one intron, and 3′- and 5’-UTR are white boxes, of the targeting vector and of the *Rab39b* recombinant locus for the generation of *Rab39b* Knock Down (KD) by homologous recombination. Neo is neomycine cassette. BamHI and NcoI, restriction enzymes used. S1 and S2, Southern blot probes. FOR, Lox2 and 3’UTR, PCR primers for *Rab39b* KD genotyping. **(B)** Southern blot shows the identification of 1D3 and 2A3 recombinant clones. The 18Kb fragment for the wild type (WT) allele or 5.4Kb band for the recombinant allele were detected by S1 probe after BamHI digestion; the 6.7 Kb band for the WT allele or 4.4 Kb band for the recombinant allele were detected by S2 probe after NcoI digestion. **(C)** Representative western blot and **(D)** quantification of RAB39B total protein expression in relative pixel intensity normalized on the intensity of the calnexin band (1D3 = 4, 2A3 = 3). **(E)** Representative image of *Rab39b* WT and KD littermate mice. Scale bar 2 cm. **(F)** Body weight in grams (*n* = 10). **(G)** Representative image of *Rab39b* WT and KD brains and **(H)** quantification in grams (*n* = 3). Data are presented as mean ± SEM. Student’s *t*-test was used in (D). ^*^P < 0.5; ^*^^*^P < 0.01; ^*^^*^^*^P < 0.001.

Two recombinant clones (1D3 and 2A3) were obtained by homologous recombination in embryonic stem cells (Fig. 4A), carrying the insertion of a unique lox *P* site into the 3’-UTR, as revealed by Southern Blot (Fig. 4B). Male chimera mice were generated and crossed with C57BL/6 N female mice to have a F1 generation of heterozygote females. *Rab39b* mutated allele showed a hypomorphic mutation that resulted in a significant down-regulation of RAB39B protein expression in 2A3 murine line compared to WT (*Rab39*b KD; 2A3-*Rab39b* WT vs KD Student’s *t*-test *P* = 0.0008; Fig. 4C and D). Backcrosses into C57BL/6 N genetic background were done, and from the sixth-generation (N6) animals were used for the experiments described. *Rab39b* KD male mice were viable and fertile and the mutant alleles were transmitted in the expected Mendelian segregation ratio of an X-linked gene. Moreover, *Rab39b* KD male mice were indistinguishable from WT littermates in the body weight that increment during development (Fig. 4E and F) and in the brain weight (Fig. 4G and H) compared to WT.

**Figure 5:**
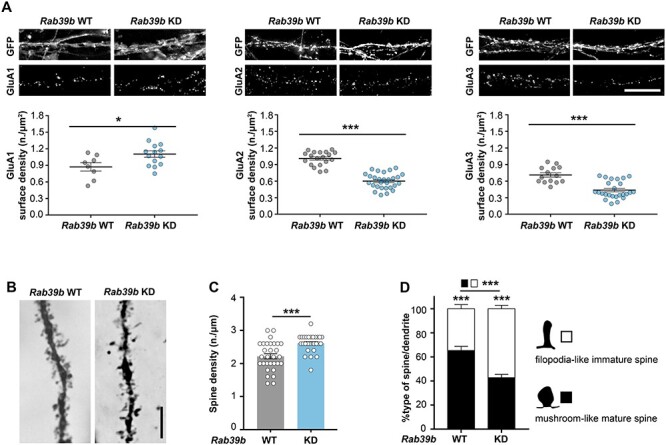
RAB39B down-regulation alters AMPAR-trafficking and spine maturation. **(A)** Representative images of immunofluorescence of GluA1, GluA2 and GluA3 surface labelling in 14DIV GFP-transduced *Rab39b* WT and KD primary hippocampal neurons. Graphs indicate the AMPAR subunits neuronal surface expression density (GluA1: WT = 8, KD = 14; GluA2 WT = 19, KD = 28; GluA3: WT = 14, KD = 25 images from a minimum of three experimental replicates), expressed in number of positive puncta/μm^2^. Scale bar 10 μm. **(B)** Representative images of apical dendrites from CA1-hippocampal area of 30 days old *Rab39b* WT and KD (*n* = 3) hippocampal slices, stained with Golgi impregnation. Scale bar 5 μm **(C)** Quantification of spine density expressed in number of spine-heads/μm. **(D)** Quantification of partitioning of spine morphology along a dendrite expressed in % of type of spine/dendrite. Data are presented as mean ± SEM. Student’s *t*-test was used. ^*^P < 0.05; ^*^^*^P < 0.01; ^*^^*^^*^P < 0.001.

Similar to the *Rab39b* KO mouse results (25), AMPAR subunit maturation and surface expression in *Rab39b* WT and KD primary hippocampal neurons were impaired (Supplementary Material, Fig. S1 and Fig. 5A). Consistent with what we showed in *Rab39b* KO mice, 30-day-old *Rab39b* KD brains revealed increased spine density compared to WT brains (Mann–Whitney U test *P* < 0.0001; Fig. 5C). Moreover, the analysis of spine morphology showed that *Rab39b* WT dendrites presented an increase in the % mushroom-like mature spines compared to the % filipodia-like immature spines *(Rab39b* WT %mature vs. %immature Mann–Whitney U test *P* < 0.0001; Fig. 5D), whereas the *Rab39b* KD spine morphology was significantly impaired, showing an opposite ratio between mature and immature spines compared to WT, in favour of immature dendritic spine structures (*Rab39b* KD %mature vs. %immature Mann–Whitney U test *P* < 0.0001; Fig. 5D).

All of these data suggest that downregulation of the total amount of RAB39B protein is able to perturb RAB39B-mediated trafficking, impacting neuronal spine development and functioning.

### RAB39B downregulation leads to social and cognitive deficits

**Figure 6:**
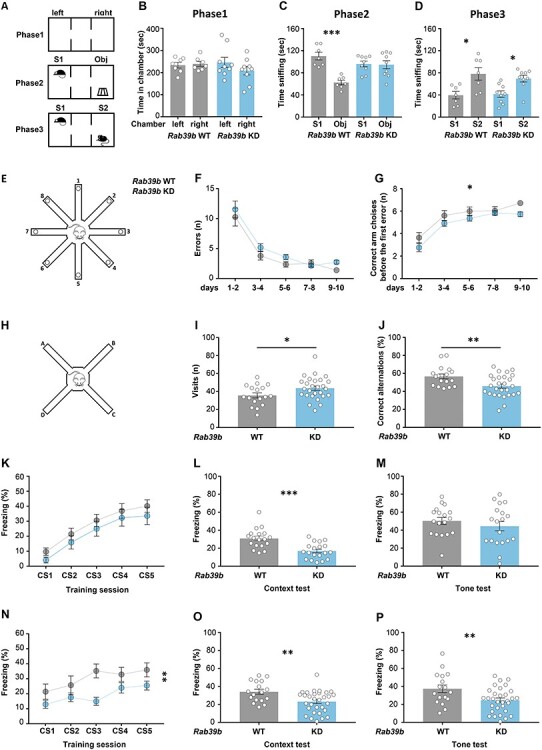
*Rab39b* KD mice show cognitive impairments. **(A)** Pictogram of 3-chamber sociability test. **(B)** Time in chamber in seconds during the phase1, **(C)** time spent sniffing the stranger1 (S1) or the object (Obj) during phase2 and **(D)** time spent sniffing stranger1 (S1) and the stranger2 (S2) in phase3 of sociability test was reported. **(E)** Pictogram of eight-arm radial maze apparatus. **(F)** Number of errors and **(G)** number of correct arm choices before the first error during the 8-arms radial maze test were presented. Dashed line is the chance level performance of 5.5 correct successive arm visits. **(H)** Pictogram of spontaneous alternation apparatus. **(I)** Number of visits and **(J)** the % of correct alternations during spontaneous alternation task were showed. **(K-M)** Delay and **(N-P)** trace fear conditioning protocols. **(K, N)** % of freezing elicited by repeated exposure to the conditioned stimulus (CS) during the training session. Pictograms on the graphs represents **(K)** the delay fear conditioning protocol where the 15 s tone (CS, black box) is superimposed with the foot shock (lightning) for the last 2 s, and **(N)** the trace fear conditioning protocol where the CS and the foot shock are separated by 15 s trace (white box). **(L, M, O, P)** % of freezing during memory test, 24 h after training, **(L, O)** for context, and **(M, P)** for tone. Data are presented as mean ± SEM. Mann–Whitney U test was used for (**A–D**) and ANOVA for (**E**–**P**). ^*^P < 0.05, ^*^^*^P < 0.01, ^*^^*^^*^P < 0.001.s

The nonstop mutation found in the two probands caused ASD, severe ID and poor motor coordination. To assess the impact of RAB39B downregulation on behavioural performance, we performed a battery of tests on 90-days old (P) *Rab39b* WT and KD male mice (Fig. 6).

#### Social and repetitive behaviours

The three-chamber sociability test assesses cognition in the form of general sociability and is used as a recognized task for ASD (28). During phase 1, the preference for left or right chambers was excluded by analyzing the time spent in each chamber (Fig. 6A and B and Supplementary Material, Table S1). During phase 2, while the *Rab39b* WT mice spent more time sniffing stranger 1 (S1) than the object (obj) (Mann–Whitney U test *P* = 0.0006; Fig. 6C), *Rab39b* KD mice spent the same amount of time investigating the S1 and obj (Fig. 6C), indicating impaired social behaviour. During phase 3, *Rab39b* WT and KD mice sniffed significantly more S2 than S1 (Mann–Whitney U test *Rab39b* WT *P* = 0.02, *Rab39b* KD *P* = 0.04; Fig. 6D), suggesting intact social recognition.

Because mouse social behaviour depends on an intact sense of smell, an olfactory-guided foraging task was performed without finding alterations in *Rab39b* KD compared to WT (Supplementary Material, Table S1).

We also investigated self-grooming, a complex innate behaviour in rodents, as a measure of repetitive behaviours (29). *Rab39b* WT and KD mice were video-tracked in different mazes to assess a possible self-grooming increase, which was also dependent on the environment. No differences between genotypes were found (Supplementary Material, Table S1).

#### Emotional and explorative behaviours

Dark&light and emergence tests were performed to assess anxiety-like behaviour and emotional and explorative behaviours (30). *Rab39b* KD mice showed behaviour comparable to that of WT mice when analyzing the variables assessing anxiety-like behaviour, such as time spent in the dark compartment in the dark & light test and time spent in the home box in the emergence test. Additionally, locomotor activity scores—the distance travelled and speed—did not show significant genotype-dependent differences. An absence of motor coordination deficits was also observed in the rotarod and gait tests.

A novelty test was performed to assess explorative, anxiety- and fear-related behaviours other than curiosity toward novelty (30). *Rab39b* KD mice were indistinguishable from WT mice in all analyzed variables (Supplementary Material, Table S1).

#### Spatial, working and associative memories

The water maze was performed to assess alterations in spatial memory (31). No differences were observed between genotypes in the time to reach the hidden platform, in the pathway, in the speed or in the annulus crossing, suggesting an intact spatial memory (Supplementary Material, Table S1).

The eight-arm radial maze (32) (Fig. 6E–G) and spontaneous alternation (33) (Fig. 6H–J) tests were used to investigate spatial and short-term working memories, respectively. In the eight-arm radial maze, *Rab39b* WT and KD mice showed a significant decline in the number of total errors over the 10 days of training, reflecting their ability to patrol the maze, without a difference between genotypes (Fig. 6F and Supplementary Material, Table S1). However, the *Rab39b* KD mice significantly differed from WT mice in the correct arm choices before the first error over 10 days of the test (ANOVA genotype effect × time F[1,42] = 5.71, *P* = 0.021; Fig. 6G). A similar deficit was observed in the spontaneous alternation task, where *Rab39b* KD mice made significantly more visits (ANOVA genotype effect F[1,42] = 4.51, *P* = 0.04; Fig. 6I), decreasing the percentage of correct alternations compared to WT mice (ANOVA genotype effect F[1,42] = 8.47, *P* = 0.006; Fig. 6J).

#### An auditory fear conditioning test was performed to assess associative learning

Two protocols were performed, differing in the training sessions: in delayed fear conditioning (DFC), the unconditioned stimulus (US) was superimposed on the last two seconds of the conditioned stimulus (CS) (Fig. 6K-M), and in trace fear conditioning (TFC), US and CS were separated by a 15-second duration (Fig. 6N-P) (34). In the training session of DFC, no difference between genotypes in the percentage of freezing was detected (Fig. 6K and Supplementary Material, Table S1). During context memory, 24 h after the training session, *Rab39b* KD mice significantly differed from WT mice (ANOVA genotype effect F[1,35] = 18.1, *P* = 0.0001; Fig. 6L). No differences were observed between genotypes for the tone memory test (Fig. 6M and Supplementary Material, Table S1).

In TFC, *Rab39b* KD mice froze significantly less than WT during conditioning (ANOVA genotype effect × time *F*[1,46] = 8.42, *P* = 0.006; Fig. 6N) as well as in the consequent context and tone test sessions (context: ANOVA genotype effect *F*[1,46] = 9.29, *P* = 0.004; tone: ANOVA genotype effect *F*[1,46] = 7.68, *P* = 0.008; Fig. 6O-P).

In conclusion, these data showed that the downregulation of RAB39B leads to a specific behavioural deficit affecting social interaction, working and associative memories.

## Discussion

In this study, we report a nonstop mutation in the *RAB39B* gene that abolishes the canonical stop codon, resulting in an extended reading frame with 21 codons of unknown function. As is usual for XLID, the clinical features associated with RAB39B mutations are variable and difficult to predict. After our initial description (15) of two families with RAB39B variants and moderate to severe ID associated with macrocephaly (family X, 6 cases), seizures (family MRX72, 3 cases), and ASD (family MRX72, 1 case), only a few other cases have been reported in the literature (24,35,36). Wilson et al. (18) reported three brothers with RAB39B deletion, severe ID, and typical parkinsonism that developed in one of them, adding RAB39B-related disease to the group of synucleopathies. A role of RAB39B in parkinsonism has been subsequently described in several cases, usually, but not exclusively, in patients with RAB39B deletions/loss-of-function mutations (19,20,23,37–39). On the other hand, an increased RAB39B dosage seems to be the most likely candidate for the neurodevelopmental delay and neurobehavioural disturbances of Xq28 duplication syndrome (17,40). Despite this evidence, no clear RAB39B genotype–phenotype correlations can be established (Table 2). This could be due to the limited cases reported in the literature or, more likely, to the influence of other genetic and/or environmental factors. Therefore, in addition to identifying new cases, their complete and accurate clinical characterization is very important.

**Table 2:** *RAB39B* variants and clinical phenotypes. ND: RAB39B protein characterization not determined; ID: Intellectual Disability; ASD: Autism Spectrum Disorder; PD: Parkinson’s Disease; §: mutation described in the present paper

Pathogenic variants	Molecular consequence	Clinical phenotypes
*Point mutations*		
c.21C > A; p.Y7X	Nonsense mutation; no protein	ID, ASD, epilepsy, macrocephaly (15)
c.215 + 1G > A	5’splice site mutation; no protein	ID, ASD, epilepsy, macrocephaly (15)
c.503C > A; p.T168K	Missense mutation; down regulation	ID, early-onset PD (18,39)
c.574G > A; p.G192R	Missense mutation; altered intracellular localization	PD (20)
c.428C > G; p.A143G	Missense mutation; ND	PD (20)
c.557G > A; p.T186X	Nonsense mutation; no protein	ID, early-onset PD (19)
c.559G > T; p.E187X	Nonsense mutation; no protein	ID, ASD, macrocephaly (24)
c.640 T > C; p.^*^214Gext^*^21	Nonstop mutation; down regulation	ID, ASD, motor problems§
*Deletion/duplication*		
c.624_626delGAG; p.R209del	Deletion; ND	PD (20)
c.432delA; p.T145Tfs^*^3	Deletion; no protein	ID, early-onset PD (37)
c.258_260delTCT; p.L88del	Deletion; ND	ID (36)
c.436_447del; p.G146_Y149del	In-frame deletion; ND	ID, ASD, epilepsy, macrocephaly (35)
c.536dupA; p.E179fs^*^48	Duplication; ND	Juvenile PD (22)
c.137dupT; p.S47Lfs^*^44	Duplication; no protein	ID, early-onset PD (23)
c.371delA; p.K124Sfs^*^10	Deletion; no protein	ID, early-onset PD (23)
*Genomic deletion/duplication*		
0.5 Mb dul Xq28	Copy-number gain; over expression	ID, behavioural problems, speech impairment (17)
45 kb del	Gene deletion; no protein	ID, early-onset PD (18)

If we consider the cases reported here (Table 1), it is well confirmed that RAB39B downregulation is associated with severe ID/cognitive impairment and significant behavioural abnormalities (level three severity using ASD standardized tests). It should be noted that although ASD has been recognized in fewer cases than ID, in many patients, the diagnosis of ASD could have been masked due to the presence of severe/moderate ID symptoms. For this reason, even when mutations of RAB39B are identified in adult patients referred to clinicians for parkinsonism, it is important to study the presence of symptoms of ASD.

Although seizures have been reported in a patient with a RAB39B mutation (15), our family did not have any medical history of seizures, nor had electroencephalogram (EEG) abnormalities ever been documented. No particular facial dysmorphisms were evident; however, the two brothers appeared very similar, both presenting elongated facies with a high forehead and large ears. There were no early signs of parkinsonism, but mild hypotonia, poor motor coordination and awkwardness were found on neurological examination. This evidence lends support to the hypothesis that RAB39B could play a more general role in neuromotor development, not limited to parkinsonism, stressing the importance of studying the pathogenic mechanisms that could be involved.

In a heterologous cell system, we demonstrated that the mutation negatively impacts RAB39B protein stability, resulting in increased protein degradation that results in a 70% protein downregulation. It is conceivable that the increased degradation rate of RAB39B ter214Q could be ascribed to protein elongation, which modifies the intrinsic characteristic of the C-terminus of RAB39B. Indeed, the C-terminus of an RAB GTPase is marked with the CXC geranylgeranylation site, which is necessary for regulated tail anchoring to the membrane vesicle, permitting the GTPase to be correctly localized and to execute its function (26). The presence of an alpha-helix organized hydrophobic stretch of aas juxtaposed to the geranylgeranylation site might dramatically affect its functionality, inhibiting the transient lipid anchoring of RAB39B or stably anchoring the protein to vesicle membranes through a transmembrane insertion or by adsorption to the membrane. This might block functional recycling of RAB39B, promoting its partial degradation (19), a topic that deserves future investigation. The remaining RAB39B was functional and able to drive AMPAR trafficking *via* PICK1 bridging in a heterologous cell system. However, in a mouse model with RAB39B protein downregulation, we confirmed the tight correlation between AMPAR surface modulation and dendritic spine reorganization, showing that *Rab39b* KD hippocampal dendritic spines are increased in density compared to WT hippocampal dendritic spines, and their shape prominently remains immature filopodia-like.

Finally, alterations in the AMPAR composition and dendritic spine maturation affect behavioural performance in a disease-specific manner. The behavioural assessment of *Rab39b* KD mice revealed normal anxiety-like, exploratory and motivational behaviour. However, the analysis of learning and memory capabilities revealed that *Rab39b* KD mice were slightly impaired in working memory tasks, as reflected in the 8-arm radial maze and the spontaneous alternation test, and were unable to form associative memories in the trace fear conditioning task. Their social behaviour was compromised but their social recognition was intact, as shown by the 3-chamber sociability test. Indeed, *Rab39b* KD mice spent the same time sniffing a never-met mouse intruder and a novel object, but they were able to recognize a new never-met mouse intruder compared to the already known mouse. This result contradicts the *Rab39b* knockout mouse social behaviour described by Zhang et al. (41), and these differences may be ascribed to the intrinsic execution of the test. In our protocol, we investigated pure social behaviour, not presenting the mouse or the object during the habituation phase (phase 1), to analyze the ability of the mouse to discriminate and choose between a never-met mouse and a never-met object.

Moreover, the comparison among all *Rab39b* mouse models generated, detailed in Table 3, revealed that depending on the neurodevelopmental stage analyzed, on the intrinsic execution of behavioural tests and on the murine genetic background, it is possible to highlight different RAB39B functions.

**Table 3:** Comparison of *Rab39b* murine models. Alterations are referred to WT littermate mice. KO: Knock Out; KD: Knock Down; PND: post-natal days; E: embryonic stage; CDS: Coding sequence; SNpc: Substantia Nigra pars compacta; SNpr: Substantia Nigra pars reticolata; VTA: Ventral tegmental area; CPu: caudate putamen; PSD post synaptic density; NPC: neuronal precursor cell; TH: Tyrosine Hydroxylase; eEPSC: evoked Excitatory Post-Synaptic Currents; mEPSC: miniature EPSC; LTP: Long Term Potentiation; §: mouse model described in the present study

Model	Generation strategy	Genetic evidences/alterations	Phenotypical alterations	Molecular alterations	Behavioural phenotype
*Rab39b* KO (39)	- CRISPR/Cas9 technology—C57BL/6 N	- NM_175122.6:c.-24_235del—NM_175122.6:c.-4_230del ${\rightarrow}$ No protein	- Not investigated	- Not investigated *Authors report RAB39B localization in cortex and hippocampus, SNpc, SNpr and VTA, but not in CPu and thalamus*	- Not investigated
*Rab39b* KO (41)	- CRISPR/Cas9 technology—C57BL/6 N	397 bp Deletions in *Rab39b* exon 2 ${\rightarrow}$ No protein	PND20 KO ↑ brain perimeter and weight ↓ body weight ↑ brain/body weight ratio ↑ hemisphere length and overall cerebral cortex areas E18.5 KO ↑ hemisphere length and overall cerebral cortex areas	E14.5, E16.5, E18.5 KO ↑ numbers of neurons at different cortical layers ↑ NPC pool E14.5 KO NPCs ↑ PI3K–AKT–mTOR signalling activity	PND60 KO—normal anxiety and mobility—social memory deficits but normal sociability—no repetitive behaviour deficits—motor learning deficits but normal motor coordination and balance
*Rab39b* KO (46)	- TALEN-technology—C57BL/6 J	‘GT’ loss at CDS sites 106–107 ${\rightarrow}$ No protein	PND60 KO ↓ body weight ↑ brain/body weight	PND60 KO *CA1-hippocampus* ↓ spine density ↓ PSD length and width ↓ GluN1, GluN2A, GluN2B in PSD fraction ↓ eEPSC slopes, LTP, NMDA/AMPA receptor response ratios, AMPA-eEPSC amplitudes ↓ autophagic flux *Midbrain* ↑ TH and ↓ dopamine levels	PND60 KO—reduced anxiety—Impaired recognition memory—impaired short-term working memory—impaired spatial memory—impaired social novelty recognition—compromised motor skill learning
*Rab39b* KO (25)	CRISPR/Cas9-mediated strategy—C57BL/6 N	Deletion of 14 bp in CDS site 28 and insertion of 10 bps in CDS site 38 ${\rightarrow}$ No protein	PND7 to 91 KO ↓ 10% body weight PND20 and 90 KO—normal brain weight and dimensions—normal organ weights ↓ volume of abdominal adipose tissue at PND90	PND30 and/or 90 KO *CA1-hippocampus* ↑ dendritic spine density ↑ filopodia-like immature spines ↓ mushroom-like mature spines *Cortical slices* ↓ AMPA-mEPSC frequency and decay time ↑ rectification index in cortical slices *Cultured hippocampal and cortical neurons* ↓ GluA2/GluA3 AMPA receptor secretory pathway and cell surface expression ↑ dendritic spine density ↑ filopodia-like immature spines ↓ mushroom-like mature spines ↑ spine dynamic	PND90 KO—increased activity—increased curiosity towards novelty—normal spatial memory—impaired short-term working memory—impaired associative memory
*Rab39b* KD §	Homologous recombination-mediated strategy—C57BL/6 N	Insertion of a unique lox P site and neomycin-resistance cassette into the 3’-UTR ${\rightarrow}$ Protein downregulation	PND7 to 91 KD—normal body weight—normal brain weight at PND30	PND30 KD *CA1-hippocampus* ↑ dendritic spine density ↑ filopodia-like immature spines ↓ mushroom-like mature spines *Cultured hippocampal neurons* ↓ GluA2/GluA3 AMPA receptor secretory pathway and cell surface expression	PND90 KD—Impaired social behaviour and normal social recognition—normal sense of smell—no repetitive behaviours—normal anxiety-like behaviour—normal motor coordination—normal spatial memory

In summary, we reported a novel *RAB39B* nonstop variant that segregates in a family with a clinical phenotype including ASD, ID and poor motor coordination. We demonstrated the pathogenicity of the RAB39B mutation that leads to RAB39B protein instability and its downregulation. In turn, its downregulation affects AMPAR trafficking and dendritic spine morphogenesis, resulting in ASD/ID-like behavioural performances in the *Rab39b* KD model, similar to the recently published *Rab39b* KO mice (25).

Nevertheless, we are aware that it would have been more appropriate to generate a knock-in mouse model carrying the exact same human mutation, but already having a mouse that mimics the final effect of the mutation, we evaluated its molecular, morphological and behavioural phenotype. The behavioural assessment of *Rab39b* KD mice reported in this study indicates they mimic the clinical features observed in the two affected brothers. The ultimate conclusion is that as far as it is possible to draw a parallel between human and murine phenotypes, the reported characterization of the *Rab39b* KD mouse and its validity could be of great interest for further evaluation.

## Materials and Methods

### Clinical assessment

Patients underwent to a complete clinical assessment, including anamnesis, physical and neurological examination, clinical observation and psycho diagnostic tests administration.

ASD diagnosis was made by expert neuropsychiatrists based on clinical judgment and Diagnostic and Statistical Manual of Mental Disorder, Fifth Edition (DSM-V) criteria, and supported by the Autism Diagnostic Observation Schedule (ADOS) and the Autism Diagnostic Interview Revised (ADI-R).

Intellectual functioning was measured using Merrill-Palmer-Revised Scales of Development (M-P-R), a standardized test including toy-based activities for children from 1 month to 6.5 years, since the poor collaborations of our patients impeded the use of the other cognitive tests.

Adaptive behaviour was assessed using Vineland Adaptive Behaviour Scale-Second Edition (Vineland-II) a semi-structured interview for individuals from birth to adulthood used to evaluate personal and social skills required for everyday living.

### Whole exome sequencing

Informed written consent and DNA from peripheral blood was collected from the probands and parents. The Whole Exome Sequencing analysis was performed Ion Torrent Next Generation Sequencing Platform: briefly, 100 ng high-quality genomic DNA was used to prepare library, according to the manufacturers’ instructions (Ion AmpliSeq™ Exome RDY Library Preparation Kit, Thermo Fisher Scientific, Inc.); sequencing was performed on the Ion Torrent Proton Sequencer (Thermo Fisher Scientific, Inc.) and sequencing data were processed using Ion Torrent platform-specific pipeline software, Torrent Suite v5.6 and then analyzed with Ion Reporter™ 5.6 Software (Annotate Variants Workflow, Reference: hg19). Sequence was numbered according to the *RAB39B* cDNA NM_171998 and protein NP_741995. The *RAB39B* mutation c.640 T > C and segregation analysis was confirmed with Sanger sequencing. The nonstop mutation p.(^*^214Qnext^*^21) will be called from here RAB39B ter214Q for simplicity.

### Mutated cDNA cloning

Total RNA from patient was extracted from blood, collected with PAXgene Blood RNA tubes (Quiagen) and processed by Maxwell 16 LEV simplyRNA Blood Kit (promega), by adapting the standard protocol following the kit application notes. RNA was reverse transcribed with M-MLV enzyme (Invitrogen). *RAB39B ter214gln* cDNA was amplified with specific primers HindR39b_F/BamHIRab39bter214Q_Rev.

(HindR39b_F: 5’-GGACAAGCTTATGGAGGCCATCTGGCTG-3′; BamHIRab39bter214Q_Rev: 5’-CCGGGGATCCCTATTTATTTCTCTTACTTTTCAG-3′) and cloned in frame in HindIII and BamHI sites of pCMV2-FLAG plasmid (Sigma-Aldrich). pFLAG is referred to pCMV2-FLAG plasmid (Sigma-Aldrich) and pFLAG*-RAB39B* WT was constructed as previously described (15).

### COS7 cell cultures and related biological and biochemical assays

COS7 cell culture and plasmid transfection—performed in equimolarity—were done as in (14). Transfected COS7 cells total RNA purification (TRIzol reagent—Invitrogen, #15596018), reverse transcription and Real-time PCR was performed as described in (15).

Transfected COS7 cells total protein isolation was performed lysing cells two days after transfection in LYSIS buffer (01% SDS, 2 mM EDTA, 10 mM Hepes pH 7.4). Cells were rotated at 4°C for 1 h before centrifuging at 12500 *g* for 20 min to pellet debris, boiled at 95°C for 5 min and syringed 5 times.

Treatments were done the day after transfection with 2 μM MG132 (Calbiochem, # 474790) or 20 μM Leupeptin (Sigma, #L2884) for 16 h before lysis; equal volume of DMSO or methanol used for dissolving MG132 and Leupeptin respectively was used as control. Treatment with 1 mM cycloheximide (Sigma, #C1988) was done the day after transfection for 1, 3, 6 and 9 h before lysis.

Western blot experiments were performed by loading 30 μg of total protein extracts onto a 12% polyacrilamide gel, then transferred on a nitrocellulose membrane by using TurboBlot technology (Biorad). Anti-RAB39B (1:500) (15) and anti-FLAG (1:1000, Sigma-Aldrich, #F7425) antibodies were used to detect the presence of FLAG-RAB39B, and anti-Calnexin antibody (1:10000, Sigma-Aldrich, # C47312) was used to normalize the total amount of protein. Chemidoc technology (Biorad) was employed to detect chemo-luminescence signal. Analysis was done using ‘Analyse gel’ plugin of ImageJ software (NIH, Bethesda, MD, USA).

Standard immunofluorescences were performed as in (14). Pictures of transfected COS7 cells were captured using Leica TCS SP8 SMD FLIM Laser Scanning Confocal equipped with a HC PL APO CS 2 63X (NA 1.4) Oil-immersion objective. Multiple focal planes with z-spacing of 0.4–0.5 μm were flattened by ImageJ maximum projection. COS7 cell morphology was detected by ‘polygon selection measurement’ plugin of ImageJ software (NIH, Bethesda, MD, USA). TIRF images, setting 110 nm as the distance from the coverslip, were acquired using Leica SR GSD 3D TIRF microscope (Leica). Total fluorescence of TIRF-GluA2 signal normalized on corrected total fluorescence of epifluorescence-RAB39B signal was quantified by ImageJ software. Correction of total fluorescence is expressed as: (integrated density—considered area) × mean fluorescence background readings.

For presentation of images, contrast was enhanced by linear methods by using ImageJ analysis software (NIH, Bethesda, MD, USA).

### Animals

Experiments were done in accordance with animal protocols approved by the ‘Institutional Animal Care and Use Committee (IACUC)’ (IRCCS San Raffaele Scientific Institute, Milan, Italy) and by the Italian National Ministry of Health (IACUC ID 652, 653), following the guidelines established by the European Community Council Directive D.L. 26/2014 on the use of animals in research (86/609/EEC). We minimize animal suffering and we used only the number of animals necessary to produce reliable results. Animals were maintained on a 12 h light/darkness cycle, inverted cycle was applied for behavioural studies. Food pellets and water were available *ad libitum*, unless stated otherwise.

### Generation and characterization of *Rab39b* knock down model

The mouse *Rab39b* gene mapped to the mouse X chromosome in XA7.3 occupying a region of 6187 bps (NC_000086.7). It is composed by two exons of 215 and 427 base pairs (bp), respectively, spaced out by one 2785 bp intron. The 5′- and 3′- untranslated regions (UTR) flanking the two exons are 224 and 2536 bps, respectively, long.

*Rab39b* knock down (KD) mouse was generated with the aim to obtain a *Rab39b*-conditional knock out mouse by homologous recombination. The targeting vector carrying the BAC-isolated isogenic C67Bl/6 N *Rab39b* gene sequence, was constructed using pFLRT plasmid as follow: the 5′ arm consisted on a 3119 base pairs (bps) fragment containing the 119 bps of 5’UTR and the full sequence of the first exon (215 bps) followed by the intron (2785 bps); *LoxP* sites were inserted, respectively, at the beginning of the second exon and 79 bps from the STOP codon, in order to delete the second exon and the initial segment of 3’UTR of *Rab39b* gene; the neomycin-resistance (neo) cassette, driven by mouse Pgk1 promoter and flanked by *Frt* sites, was inserted immediately downstream of the second *LoxP* site; the 3’arm of the targeting vector consisted in 1276 bps containing the fragment of 3’UTR.

After linearization with unique NotI restriction site, targeting vector was electroporated in embryonic stem (ES) cells. About, 600 G418-resistant colonies were analyzed by southern blot using 5′ and 3′ external probes. The 5′ flanking probe, S1 (PCR product from primers 5’ARM10F, 5′-GCATGTTTGAGCATGGAAACCCG-3′ and PROBE5’REV, 5′-CCTGAAGCAAGGTAGGCCCAGG-3′) detected a band of 18 Kb for the WT allele and 4.7 Kb for the recombinant allele, following the cut with the restriction enzyme BamHI. The 3′ flanking probe, S2 (PCR product from primers PROBE3’FOR, 5′-GGATTGTAGCCAGTTCAGCTAGG-3′, and PROBE3’REV, 5′-CCACTTATTGCAGTGACAACAGC-3′) displayed a band of 6.7 Kb for the WT allele and 4.4 Kb for the recombinant allele, following the cut with restriction enzyme NcoI. However, southern blot showed a band of 5.4 Kb instead of the expected 4.7Kb following the hybridization with S1 probe, identifying two ES clones where the recombination occurred inside the region surrounded by *LoxP* site, leading to ES clones containing only the second *LoxP* site and neomycin cassette in the 3’UTR.

Two homologous recombinant clones were injected into 129B6 F1 blastocysts by standard methods. Chimeric mice from both clones were crossed to C57BL/6 N female mice.

Genotypes were determined by PCR from DNA-tail using combination of three primers: For (5′- GAGGTTATCAAATCAGAGAGGAG-3′), Lox2 (5′- TCCCGGGGATCGATCCGGAA-3′) and 3’UTR (5′- CCCAGACCCTTAATATGTGGTC-3′). For and 3’UTR amplified a 322 bps fragment of the WT allele, For and Lox2 amplified a 309 bps band from the transgenic allele.

Total protein isolation of murine brains were obtained by lysing tissues in homogenation buffer [320 mM saccarose, 5 mM Hepes pH 7.4, 2 mM EDTA, 10 mM sodium fluoride, 1 mM sodium orthovanadate, 1 mM β-glicerophosphate, 1× protease inhibitor cocktail (Sigma-Aldrich, #P8340)], then lysates were incubated 10 minutes at +4°C on rotation and centrifuged 13 500 g 20 minutes at +4°C. Western blot experiments were performed by loading 50 μg of total protein extracts onto a 12% polyacrilamide gel, then transferred on a nitrocellulose membrane as standardized procedure. Anti-RAB39B (1:500) (15) was used for detecting the presence of RAB39B, and monoclonal anti-Calnexin (1:10000, Sigma-Aldrich, # C47312) was used for normalizing the total amount of protein. Analysis was done using ‘analyse gel’ plugin of ImageJ software (NIH, Bethesda, MD, USA).

Hippocampal total RNA isolation, reverse transcription and real-time PCR was performed as described in (14).

### Hippocampal cultured neurons, lentiviral transduction and image acquisition

Primary neuronal cultures were prepared as in (42), 150 000 neurons/Ø24 mm-coverslip were plated. Neurons were transduced (MOI1) with lentiviral particles expressing GFP at 2 day in vitro (DIV) as previously described (15).

Immunofluorescences to visualize the intracellular distribution and the surface expression of AMPAR subunit were performed as described in (14). Pictures of hippocampal neurons were captured using DeltaVision microscope (Applied Precision) equipped with a 60× or 100× objectives. Multiple focal plans with z-spacing of 0.20 μm were deconvolved and flattened by maximum projection. ‘Gran filter’ plugin of ImageJ Analysis Software (NIH, Bethesda, MD, USA; setting the size of circle from 1 to infinity) was used to measure AMPAR subunits density relatively to GFP area. For presentation of images, contrast was enhanced by linear methods by using ImageJ analysis software (NIH, Bethesda, MD, USA).

### Western blot and de-glycosylation assay on primary hippocampal neurons

For a detailed description, see Supplementary Information.

### Golgi staining

Golgi Staining was performed following the FD Rapid GolgiStain Kit (FD NeuroTechnologies, Columbia, MD, US). Pictures from neuronal apical dendrites in CA1 region of hippocampus were captured using AxioImager—Zeiss AxioImager M2m equipped with a 100× objective by comparing similar segments of dendrites between different cells (43). Three mice per genotype and age were analyzed, 5 dendrites per hemisphere from one slice per mouse were examined. Number of head spines was manually counted and normalized on 5 μm dendritic length measured by ImageJ analysis software (NIH. Bethesda. MD, USA). Morphology of spines was described as the ratio of spine length and spine width (44). A maturity-ratio less than 2.5 represented mature mushroom-like spine morphology, a ratio greater than 2.5 denotes immature filopodia-like spines.

### Behavioural tests and statistical analysis

#### Three-chamber sociability test

Three-chamber sociability test was performed as previously described (28). The apparatus consisted on three-chambered box with openings between the chambers. The test consisted in 3 phases of 10 minutes. Phase 1: habituation to the empty 3-chambered box. Phase 2: a never-before-met mouse (stranger 1) was inserted under a small cage into the left or right chamber and an empty small cage (object) in the right or left chamber; the positioning of stranger 1 and object was done randomly. Phase 3: the object was replaced with a new never-before-met mouse (stranger 2). The time spent investigating (sniffing) the stranger 1, the object and the stranger 2 was continuously scored. The absence of preference for left or right chamber was excluded by analyzing the time spent in chambers during the phase 1.

#### Olfactory-guided foraging task

The test was performed as described in (34). Briefly, deprived mice were habituated to a piece of milk chocolate for 8 h to explore and consume it, and after this period, mice received normal food. On test day, five trials were done and the time to find the chocolate was scored. Trial 1 consist on placing the chocolate on the surface of the bedding in the middle of a clean test cage; during trial 2–5, the chocolate was hidden under the bedding in one of each corner of the test cage.

#### Self-grooming

Self-grooming assessment was evaluated in different environment and manually scored: in the 3-chambered box, 4-arms maze and the grid floor of fear conditioning box.

#### Dark&light test

The Dark&light test (30) was performed in a 20 × 30 cm lit chamber with transparent Perspex walls (20 cm high) and open top was connected to a 20 × 15 × 20 cm plastic grey box, which was completely closed, except for the 7.5 × 7.5 cm door connecting it to the lit chamber. Illumination was by direct room light, 500 lux. Each mouse was released in the middle of the lit compartment and observed for 5 min.

#### Emergence test

The emergence test (30) was carried out into 50 × 50 cm square arenas of non-reflective aluminium (37 cm high). Illumination in the room was provided by indirect diffuse light (4 × 40 W bulbs, 12 lx). The first day of the test consisted in placing a plastic box (12 × 8 × 4 cm with an 8 × 4 cm opened door) in the mouse’s home cage. In the second day of the test, the same plastic box was placed 5 cm from the corner of the testing arena, with the opened door facing to the centre of the arena, then mouse was released in the centre of the arena for 30 min.

#### Novelty test

The Novelty test (30) was performed in the same apparatus of the emergence test. The test consisted in 2 phases of 30 min: in the first phase mouse was let free to explore the arena, during the second phase an object was introduced in the centre of the arena.

#### Rotarod test

Rotarod apparatus was composed by a horizontal rotating rod (3 cm diameter) and five mice were simultaneously placed on the rotarod apparatus at the same time, separated by large disks (Ugo Basile S.r.L., Italy). On day 1, accelerating session, the rotation speed increased every 30 s of 4 rpm. On day 2, constant speed calculated as the average of all maximum speed reached from all mice on day 1. For both days, each mouse was submitted to 5 trials with an inter-trial interval of 30 min and a trial ends when the mouse falls down or when 5 min were elapsed. The latency to fall off the rod is taken as the dependent variable for every trial.

#### CatWalk test

Gait was assessed by using the Catwalk XT Gait Analysis System (Noldus Information Technology, Asheville, NC, USA). Mice were allowed to freely ambulate along an illuminated glass plate within a confined corridor (L 50 × W 8 cm) in a darkened room and footprints were recorded with a high-speed camera for following analyses with CatWalk XT 10.0 software (Noldus). Three compliant (time-constrained) trials/animals were analyzed, averaged and the mean of the average/genotype calculated.

#### Water maze task

The standard hidden-platform version of the water maze was done as previously described (31). Briefly, the test included an acquisition phase (6 trials/day, for 3 days) followed by a reversal phase during which the platform was moved to the opposite position (6 trials/day, for 2 days). The trials were averaged in blocks of 2 trials for the analysis.

#### Radial maze

The 8-arms radial maze test was performed as previously described (34). The apparatus consisted of 8 arms (38-cm long, 7-cm wide) extending from an octagonal centre platform (diameter 18.5 cm) with 5-cm transparent plastic walls. The distance from the platform centre to the end of each arm was 47 cm. A cup with a food pellet was present at the end of each arm. Food-deprived mice (maintained at 85% of their free-feeding weight) were placed in the centre platform and allowed to collect pellets placed at the end of each arm for 10 min. The animals were adapted to the maze for 1 day and then tested for 10 days. For each trial, the total number of arm choices, the number of correct choices before the first error, and the total number of errors were recorded.

#### Spontaneous alternation test

The apparatus used is the same as described for the Radial maze with only four arms open. A mouse was released in the central hub of a cross maze (4 arms) and left free to explore for 10 min. Number and sequence of arm entries were recorded. A correct alternation was considered when no more than one repetition over 5 entries was made.

#### Fear conditioning

Auditory fear conditioning test (34) was performed to assess associative learning. During the conditioning, the tone becomes a conditioned stimulus (CS) able to elicit a conditioned reaction (freezing) when paired with an unconditioned foot shock stimulus (US).

Two protocols were performed, differing in the training session: in delay fear conditioning (DFC) US is superimposed to the last two seconds of the CS, in trace fear conditioning (TFC) US and CS were separated by 15 s trace. The context and the tone memory was analyzed 24 h later.

#### Video tracking and data collection

Animals were video-tracked using the EthoVision 2.3 system (Noldus Information Technology, Wageningen, the Netherlands; http://www.noldus.com) using an image frequency of 4.2/s. Raw data were transferred to Wintrack 2.4 (http://www.dpwolfer.ch/wintrack) for offline analysis.

For fear-conditioning protocols, animals were video-tracked using the ANY-maze system (Anymaze, Stoelting, Wood Dale, IL, USA).

### Statistical analysis

Data are reported as mean ± SEM. Graphs were generated and statistical analysis were performed using GraphPad Prism v7 or StatView (SAS Institute, Cary, NC, USA). Statistical analysis was specified in the text and figure legends if significant *P*-values are detected. Supplementary Material, Table S1 reported behavioural non-statistically significant *P*-values. In particular, Shapiro–Wilk normality test was performed to assess the normal distribution of data. If data violate normality test, non-parametric Mann Whitney U test un-paired *t*-test was performed. If data pass the normality test, un-paired Student’s *t*-test or analysis of variance (ANOVA), alpha value 5%, with factorial or repeated measures were used. Two-tailed *P*-values <0.05 were considered statistically significant (^*^P < 0.05, ^*^^*^P < 0.01, ^*^^*^^*^P < 0.001).

**List of abbreviations**
ASD: Autism spectrum disorder
DSM-V: Diagnostic and statistical manual of mental disorders
ID: Intellectual disability
AMPAR: α-amino-3-hydroxy-5-methyl-4-isoxazolepropionic acid receptor
ER: Endoplasmic reticulum
KD: Knock down
UTR: Untranslated region
bp: Base pairs
DIV: Days in vitro
ADI-R: Autism Diagnostic Interview-Revised
ADOS: Autistic Diagnostic Observation Schedule
M-P-R: Merill-Palmer-Revised
VABS: Vineland Adaptive Behaviour Scales-II
IQ: Intellectual quotient
EEG: Electroencephalogram
AA: Amino acid
S: Stranger
Obj: Object
TIRF: Total internal reflection fluorescence
WES: Whole exome sequencing
P: Days old
DFC: Delay fear conditioning
TFC: Trace fear conditioning
US: Unconditioned stimulus
CS: Conditioned stimulus

## Supplementary Material

Supplementary_materials_ddab320
