# Supplementary material for: Clinical characterization of a novel RAB39B nonstop mutation in a family with ASD and severe ID causing RAB39B downregulation and study of a Rab39b knock down mouse model
Source: Hum Mol Genet. 2021 Nov 11;31(9):1389–406. doi: 10.1093/hmg/ddab320 (PMC9071400; doi:10.1093/hmg/ddab320)

**SUPPLEMENTARY INFORMATION**

**Supplementary Results**

**RAB39B down-regulation impairs GluA2/GluA3 AMPAR maturation**

As previously described (1), RAB39B mediates the GluA2/GluA3 trafficking from the ER to the Golgi complex. Established that RAB39B down-regulation did not affect the total amount of AMPAR subunit proteins in *Rab39b* WT and KD neurons by western blot (Supplementary Fig. 1A), we next determined the effect of RAB39B down-regulation on AMPAR subunits secretory pathway by examining their maturation status, defined by the ratio between mature and immature isoforms (maturity ratio) following EndoH digestion (Supplementary Fig. 1B) (1). The maturity ratio of GluA2 and GluA3 significantly decreased in *Rab39b* KD compared to WT (Student’s t-test, GluA2 p=0.02, GluA3 p=0.006), while GluA1 ratio is not affected compared to WT (Supplementary Fig. 1B).

**Supplementary Figures**

**Supplementary Figure 1. RAB39B down-regulation alters AMPAR-****trafficking. (A)** Quantiﬁcation of the total protein amount of GluA1, 2 and 3 AMPAR subunits (n=3) in 14DIV *Rab39b* WT and KD primary hippocampal neuron lysates, expressed in relative pixel intensity to the housekeeping and representative western blots. Calnexin was used as housekeeping. **(B)** Quantiﬁcation of the ratio between mature (M) and immature (IM) forms of AMPAR subunits after EndoHf digestion of 14DIV *Rab39b* WT and KD primary hippocampal lysates. Lower panels are representative western blots for GluA1 (n=3), GluA2 (n=4) and GluA3 (n=3) after PNGasef (P) or EndoHf (E) digestion, ND: not-digested neurons. *p<0.05, **p<0.01, ***p<0.001.

**Supplementary methods**

**Western blot and de-glycosylation assay on primary hippocampal neurons**

Mouse primary hippocampal neurons were lysed at 14DIV in LYSIS buffer, boiled for 5 minutes and syringed 8 times. SDS-PAGEs were run with 10% poly-acrylamide gel, and then western blots were performed. Primary antibodies used were: anti-GluA1 (1:1000, Millipore, #MAB2263); anti-GluA2 (1:1000, Millipore; #MAB397); anti-GluA3 (1:1000, Alomone lab, #AGC-010). Calnexin (1:10000, Sigma-Aldrich, # C47312) was used as housekeeping.

De-glycosilation assay - EndoHf (NEB, #P0703S) and PNGasef (NEB, #P0704S) - were followed according to the manufacturer’s instructions to digest proteins, with minor modifications previously described in (1).

Bands intensity were quantified with ‘Analyze gels’ plugin of ImageJ software (NIH, Bethesda, MD, USA).

1. Mignogna, M.L., Giannandrea, M., Gurgone, A., Fanelli, F., Raimondi, F., Mapelli, L., Bassani, S., Fang, H., Van Anken, E., Alessio, M., *et al.* (2015) The intellectual disability protein RAB39B selectively regulates GluA2 trafficking to determine synaptic AMPAR composition. *Nature communications*, **6**, 6504.

| **Test** | **N:WT*vs*KD** | **Variable analyzed** | **Statistics** |
| --- | --- | --- | --- |
| **3-chamber Sociability** | 7 vs 9 | Time in chamber-habituation | Mann Whitney U test WT p=0.90, KD p=0.38 |
| **Olfactory- task** | 6 vs 5 | Time to find chocolate - surface  Time to find chocolate - hidden | ANOVA geno effect: F[1,9]=3.8, p=0.09  ANOVA geno effect: F[1,9]=1.3, p=0.29 |
| **Self-grooming** | 7 vs 9  30 vs 28  10 vs 5 | Time in self-grooming - 3-chamber  Time in self-grooming - 4-arms  Time in self-grooming - grid | ANOVA geno effect: F[1,14]=0.02, p=0.88  ANOVA geno effect: F[1,56]=1.96, p=0.17  ANOVA geno effect: F[1,13]=0.11, p=0.75 |
| **Dark and Light** | 32 vs 30 | Time spent in dark compartment | ANOVA geno effect: F[1,59]=1.36, p=0.25 |
|  |  | Distance travelled | ANOVA geno effect: F[1,59]=0.7, p=0.41 |
|  |  | Speed | ANOVA geno effect: F[1,59]=0.48, p=0.49 |
| **Emergence** | 32 vs 30 | Time spent in the home box | ANOVA geno effect: F[1,58]=0.28, p=0.60 |
|  |  | Distance travelled | ANOVA geno effect: F[1,58]=1.41, p=0.24 |
|  |  | Speed | ANOVA geno effect: F[1,57]=2.55, p=0.12 |
| **Rotarod** | 27 vs 24 | Latency to fall – acceleration  Latency to fall – constant speed | ANOVA geno effect: F[1,49]=0.51, p=0.48  ANOVA geno effect: F[1,49]=1.51, p=0.22 |
| **CatWalk** | 15 vs 13 | Stride length of right front paw  Stride length of left front paw  Stride length of right hind paw  Stride length of left hind paw  Base of front paws  Base of hind paws | ANOVA geno effect: F[1,26]=1, p=0.33  ANOVA geno effect: F[1,26]=0.47, p=0.5  ANOVA geno effect: F[1,26]=1, p=0.32  ANOVA geno effect: F[1,26]=0.13, p=0.72  ANOVA geno effect: F[1,26]=0.68, p=0.42  ANOVA geno effect: F[1,26]=0.42, p=0.52 |
| **Novelty** | 12 vs 11 | Distance travelled | ANOVA geno effect: F[1,21]=0.97, p=0.34 |
|  |  | Speed | ANOVA geno effect: F[1,20]=2.26, p=0.15 |
|  |  | Time spent in the corners | ANOVA geno effect: F[1,21]=0.48, p=0.50 |
|  |  | Distance to the object, while in the object zone | ANOVA geno effect: F[1,18]=0.48, p=0.50 |
| **8-arm Radial maze** | 14 vs 30 | Number of errors | ANOVA geno effect: F[1,42]=1.73, p=0.19 |
| **Water maze** | 21 vs 20 | Time to reach the hidden platform | ANOVA geno effect: F[1,39]=0.63, p=0.43 |
|  |  | Pathway | ANOVA geno effect: F[1,39]=1.31, p=0.26 |
|  |  | Speed | ANOVA geno effect: F[1,39]=0.38, p=0.54 |
|  |  | Annulus crossing (n) | ANOVA geno effect: F[1,39]=1.50, p=0.22 |
| **Delay fear conditioning** | 18 vs 19 | %freezing – Training session | ANOVA geno effect: F[1,35]=1.82, p=0.18 |
|  |  | %freezing – Tone | ANOVA geno effect: F[1,35]=0.82, p=0.37 |

**Supplementary Table 1. *Rab39b* KD behavioral analysis**


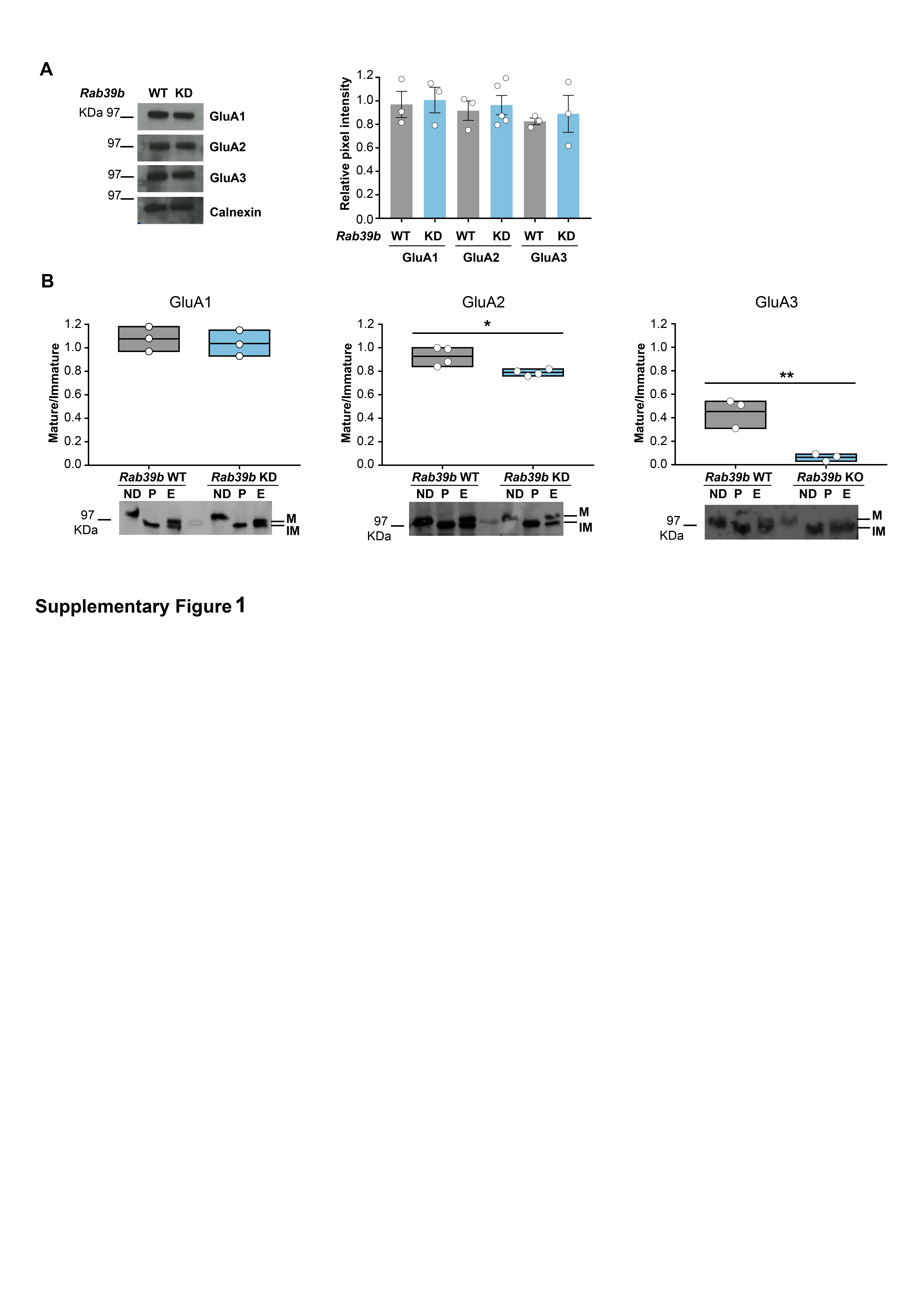

Supplement: Supplementary_materials_ddab320 [file supplementary_materials_ddab320.docx]
